# Can one overcome “unhealthy genes”?

**DOI:** 10.1038/s41525-019-0099-2

**Published:** 2019-10-02

**Authors:** Julieta Lazarte, Robert A. Hegele

**Affiliations:** Departments of Medicine and Biochemistry, and Robarts Research Institute, Schulich School of Medicine and Dentistry, Western University, London, ON Canada

**Keywords:** Risk factors, Genetics

“A book is not an isolated being: it is a relationship, an axis of innumerable relationships”―Jorge Luis Borges*, Ficciones*

Like a book, our genome is not isolated from innumerable relationships, including those with the environment and conscious lifestyle choices.^1^ Furthermore, an axis of complex interactions between individual genes underlies the phenotype at any point in time. In atherosclerotic cardiovascular disease (ASCVD), the predicted deleterious effect of single gene variant and polygenic susceptibility can be offset by external factors and counterbalancing genetic effects. Given that our experience is in the field of lipidology, we will make that the focus of this piece. For instance, individuals with heterozygous familial hypercholesterolemia (FH) most often due to loss-of-function variants in the *LDLR* gene^2^ have markedly elevated levels of low-density lipoprotein (LDL) cholesterol and are strongly predisposed to develop premature heart attacks and strokes. Observational studies show that daily statin use by FH patients reduces their ASCVD risk by 44%.^3^ Furthermore, the onset of adverse ASCVD events in FH appears to be delayed by at least two decades by attention to physical activity and moderating intake of saturated fat.^4^

The effects of a deleterious variant can also be canceled out by rare genomic variants. For instance, the phenotypic mirror image of elevated LDL cholesterol levels in FH are low cholesterol levels in heterozygous hypobetalipoproteinemia, which can result from dominant rare variants in several genes, including *APOB*, *PCSK9*, and *ANGPTL3*.^5^ In a large Utah kindred, a rare FH-causing *LDLR* variant on chromosome 19p13 segregated independently of a second rare hypobetalipoproteinemia-causing *APOB* variant on chromosome 2p24.^6^ In a nuclear family with a parent with each type of heterozygous variant, offspring could have very low or high cholesterol levels consistent with simple heterozygosity for each variant. However, some individuals also had normal cholesterol levels; this was due to either inheriting wild-type alleles from both parents, or concurrently inheriting the cholesterol-raising and -lowering variant.^6^ A similar family was reported from Holland, in which the father and mother carried, respectively, a heterozygous cholesterol-raising and -lowering variant, with some children having normal cholesterol levels due to concurrent inheritance of both rare variants.^7^ More recently, heterozygous truncating *APOB* variants were shown in Mendelian randomization studies in unrelated populations to be associated with reduced cholesterol and ASCVD risk.^8^ Such observations inspired the development of mipomersen, an antisense oligonucleotide targeting APOB that was approved in the US for treatment of increased LDL cholesterol levels in homozygous FH patients,^9,10^ although sales were discontinued in May 2018 because of safety concerns.

Similar “experiments of nature” involving rare variants of large effect have motivated development of other medications for elevated cholesterol. Mendelian randomization studies showed reductions of both LDL cholesterol levels and ASCVD risk in heterozygous carriers of loss-of-function variants in the *PCSK9* gene. This gave rise to development of both antibody and antisense strategies to reduce proprotein convertase subtilisin kexin 9 (PCSK9) levels.^11^ The monoclonal anti-PCSK9 antibodies alirocumab and evolocumab were approved for clinical use in 2015^12,13^; both drugs also significantly reduce ASCVD events such as strokes and heart attacks.^14^ These drugs are especially effective in heterozygous FH patients.^15–17^

Another beneficial drug target identified by genetics is angiopoietin like 3 protein (ANGPTL3). Mouse studies showed that deleting *Angptl3* protected against atherosclerosis,^18^ while studies in human pedigrees showed very low lipid levels in family members with recessive *ANGPTL3* loss-of-function variants.^19^ A subsequent Mendelian randomization study established that individuals with heterozygous *ANGPTL3* loss-of-function variants had reduced lipid levels and were protected from heart attacks.^20^ This encouraged the development of agents targeting ANGPTL3, including evinacumab, an investigational monoclonal antibody that strikingly reduces LDL cholesterol levels.^21^

Finally, genetic susceptibility to ASCVD most frequently results from the aggregated burden of common small effect variants, typically single nucleotide polymorphisms. The cumulative impact on ASCVD risk of numerous small effect variants per genome is quantified using polygenic risk scores (PRSs).^22^ Individuals in the highest decile of the PRS distribution have approximately threefold increased risk of ASCVD compared with individuals in the lowest decile.^23^ Because these scores appear to add prognostic information above and beyond traditional variables, they are poised to be adopted clinically. Furthermore, it appears possible to overcome polygenic predisposition to ASCVD: a study of 55,685 individuals showed that among those with the highest risk (top quintile of PRS), a favorable lifestyle was associated with ~50% lower relative risk of coronary artery disease than was an unfavorable lifestyle.^24^

Thus, for a complex condition such as ASCVD, substantial evidence shows that strong genetic predisposition, both from rare large effect variants and accumulated small effect variants, can be overcome by relatively simple interventions. These include risk factor modification, such as proper diet and increased level of activity, and where appropriate, use of established safe generic statin medications. Furthermore, understanding of how secondary genetic factors have reduced risk in predisposed individuals has led to development of several novel drugs that are either already approved or in the late stages of development. Finally, despite its unique genetic and pathogenic features, ASCVD is one example of a complex medical condition for which genetic susceptibility can be overcome.

## References

[1] Khera AV, Kathiresan S (2017). Genetics of coronary artery disease: discovery, biology and clinical translation. Nat. Rev. Genet..

[2] Berberich AJ, Hegele RA (2019). The complex molecular genetics of familial hypercholesterolaemia. Nat. Rev. Cardiol..

[3] Besseling J, Hovingh GK, Huijgen R, Kastelein JJP, Hutten BA (2016). Statins in familial hypercholesterolemia: consequences for coronary artery disease and all-cause mortality. J. Am. Coll. Cardiol..

[4] Hegele RA (1989). Clinical application of deoxyribonucleic-acid markers in a Utah family with hypercholesterolemia. Am. J. Cardiol..

[5] Hartz J, Hegele RA, Wilson DP (2019). Low LDL cholesterol-friend or foe?. J. Clin. Lipidol..

[6] Emi M (1991). Effects of three genetic loci in a pedigree with multiple lipoprotein phenotypes. Arterioscler. Thromb..

[7] van der Graaf A (2008). Familial defective apolipoprotein B and familial hypobetalipoproteinemia in one family: two neutralizing mutations. Ann. Intern. Med..

[8] Peloso GM (2019). Rare protein-truncating variants in APOB, lower low-density lipoprotein cholesterol, and protection against coronary heart disease. Circ. Genom. Precis. Med..

[9] Hegele RA, Tsimikas S (2019). Lipid-lowering agents. Circ. Res..

[10] Tsimikas S (2018). RNA-targeted therapeutics for lipid disorders. Curr. Opin. Lipidol..

[11] Rosenson RS, Hegele RA, Fazio S, Cannon CP (2018). The evolving future of PCSK9 inhibitors. J. Am. Coll. Cardiol..

[12] Schwartz GG (2018). Alirocumab and cardiovascular outcomes after acute coronary syndrome. N. Engl. J. Med..

[13] Sabatine MS (2017). Evolocumab and clinical outcomes in patients with cardiovascular disease. N. Engl. J. Med..

[14] Santos RD (2017). Genetics and molecular biology controversies on Mendelian randomization and proprotein convertase subtilisin-kexin type 9 inhibitor clinical trials: more data still necessary. Curr. Opin. Lipidol..

[15] Kastelein JJ (2015). ODYSSEY FH I and FH II: 78 week results with alirocumab treatment in 735 patients with heterozygous familial hypercholesterolaemia. Eur. Heart J..

[16] Ginsberg HN (2016). Efficacy and safety of alirocumab in patients with heterozygous familial hypercholesterolemia and LDL-C of 160 mg/dl or higher. Cardiovasc. Drugs Ther..

[17] Raal F (2012). Low-density lipoprotein cholesterol-lowering effects of AMG 145, a monoclonal antibody to proprotein convertase subtilisin/kexin type 9 serine protease in patients with heterozygous familial hypercholesterolemia: the Reduction of LDL-C with PCSK9 Inhibition in Heterozygous Familial Hypercholesterolemia Disorder (RUTHERFORD) randomized trial. Circulation.

[18] Ando Y (2003). A decreased expression of angiopoietin-like 3 is protective against atherosclerosis in apoE-deficient mice. J. Lipid Res..

[19] Musunuru K (2010). Exome sequencing, ANGPTL3 mutations, and familial combined hypolipidemia. N. Engl. J. Med..

[20] Stitziel NO (2017). ANGPTL3 deficiency and protection against coronary artery disease. J. Am. Coll. Cardiol..

[21] Gaudet D (2017). Safety and efficacy of evinacumab, a monoclonal antibody to Angptl3, in patients with homozygous familial hypercholesterolemia: a single-arm, open-label, proof-of-concept study. Atherosclerosis.

[22] Dron JS, Hegele RA (2019). The evolution of genetic-based risk scores for lipids and cardiovascular disease. Curr. Opin. Lipidol..

[23] Khera AV (2018). Genome-wide polygenic scores for common diseases identify individuals with risk equivalent to monogenic mutations. Nat. Genet..

[24] Khera AV (2016). Genetic risk, adherence to a healthy lifestyle, and coronary disease. N. Engl. J. Med..

